# Staged laparotomies based on the damage control principle to treat hemodynamically unstable grade IV blunt hepatic injury in an eight-year-old girl

**DOI:** 10.1186/s40792-016-0264-0

**Published:** 2016-11-16

**Authors:** Takashi Kobayashi, Masayuki Kubota, Yuhki Arai, Toshiyuki Ohyama, Naoki Yokota, Kohei Miura, Hirosuke Ishikawa, Daiki Soma, Kazuyasu Takizawa, Jun Sakata, Masayuki Nagahashi, Hitoshi Kameyama, Toshifumi Wakai

**Affiliations:** 1 Department of Pediatric Surgery, Niigata University Graduate School of Medical and Dental Sciences, 1-757, Asahimachi-dori, Chu-o-ku, Niigata, 951-8510 Japan; 2 Division of Digestive and General Surgery, Niigata University Graduate School of Medical and Dental Sciences, Niigata, Japan

**Keywords:** Blunt hepatic injury, Children, Damage control surgery, Transarterial embolization, Delayed hepatic resection

## Abstract

**Background:**

Severe blunt hepatic injury is a major cause of morbidity and mortality in pediatric patients. Damage control (DC) surgery has been reported to be useful in severely compromised children with hepatic injury. We applied such a technique in the treatment of a case of hemodynamically unstable grade IV blunt hepatic injury in an eight-year-old girl. This case is the first to use multimodal approaches including perihepatic packing, temporary closure of the abdominal wall with a plastic sheet, transarterial embolization (TAE), and planned delayed anatomical hepatic resection in a child.

**Case presentation:**

An eight-year-old girl was run over by a motor vehicle and transferred to the emergency department of the local hospital. Her diagnoses were severe blunt hepatic injury (grade IV) with left femoral trochanteric fracture. No other organ injuries were observed. Because her hemodynamic state was stable under aggressive fluid resuscitation, she was transferred to our hospital for surgical management. On arrival at our institution about 4 h after the injury, her hemodynamic condition became unstable. Abdominal compartment syndrome also became apparent. Because her condition had deteriorated and the lethal triad of low BT, coagulopathy, and acidosis was observed, a DC treatment strategy was selected. First, emergent laparotomy was performed for gauze-packing hemostasis to control intractable bleeding from the liver bed, and the abdomen was temporarily closed with a plastic sheet with continuous negative pressure aspiration. Transarterial embolization of the posterior branch of the right hepatic artery was then carried out immediately after the operation. The lacerated right lobe of the liver was safely resected in a stable hemodynamic condition 2 days after the initial operation. Bleeding from the liver bed ceased without further need of hemostasis. She was transferred to the local hospital without any surgical complications on day 42 after admission. She had returned to her normal life by 3 months after the injury.

**Conclusion:**

The DC strategy was found to be effective even in a pediatric patient with hemodynamically unstable severe blunt hepatic injury. The presence of the deadly triad (hypothermia, coagulopathy, and acidosis) and abdominal compartment syndrome was an indication for DC surgery.

## Background

Damage control (DC) surgery has created a paradigm shift in strategies of simultaneous definitive repair of injured organs. In DC surgery, the initial surgery is performed to control hemorrhaging and stabilize the general circulation, after which definitive surgeries are performed as needed [1, 2].

The application of DC principles is considered when a patient shows the lethal triad of hemorrhagic shock associated with acidosis, coagulopathy, and hypothermia. In children, the indication for DC surgery is considered to be the same as in adults. However, children are anatomically and physiologically different from adults in many respects. Therefore, the application of expeditious DC surgery is mandatory in children with exsanguinating hepatic and/or vascular injuries [3]. We herein report a case of an eight-year-old girl with right hepatic injury who was hit by an automobile. Her severe hemorrhagic shock was successfully managed by DC surgery.

To our knowledge, this case is the first using multimodal approaches including perihepatic packing, temporary closure of the abdominal wall with a plastic sheet, transarterial embolization (TAE), and planned delayed anatomical hepatic resection in a child.

## Case presentation

The patient was an eight-year-old Japanese girl who lived in the northern area of Japan. A large amount of snow had piled up on the sidewalk, and while she was enjoying this snow, she accidentally slid down into the road and was run over by a motor vehicle. She was transferred to the emergency department of the local hospital about 48 min after the accident. On arrival, she complained of severe upper abdominal pain with prominent abdominal distention. She was pale, and a tire mark obliquely running over the right lower abdomen was found. Although her blood pressure (BP) was maintained at 109/71 mmHg, her heart rate (HR) was increased to 157 bpm. Her respiratory rate (RR) was 30 bpm, and the percutaneous oxygen saturation on the index finger (SatO_2_) was 100%. She scored a 15 on the Glasgow coma scale (GCS). Focused assessment with sonography for trauma revealed intraperitoneal fluid collection, suggesting hemoperitoneum. Her BP decreased to 77/57 mmHg soon after admission, and fluid resuscitation with 30 ml/kg lactated Ringer’s solution was started. Her hemodynamic condition recovered with a BP of 109/75 mmHg, and her HR slightly dropped to 139 bpm.

**Fig. 1:**
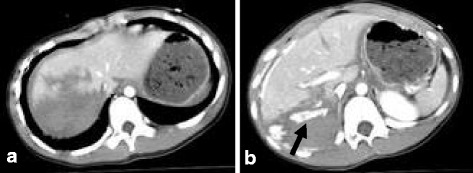
**a**, **b** Axial view of an enhanced abdominal computed tomography (CT) scan shows the presence of active extravasation (*arrow*), large devascularization of the right hepatic lobe, and hemoperitoneum

A contrast-enhanced computed tomography (CT) scan taken after she achieved hemodynamic stabilization showed a parenchymal disruption of the right hepatic lobe (grade IV) [4, 5] with extravasation of contrast material from the hepatic artery to the peritoneal cavity (Fig. 1a, b). Left trochanteric fracture of the femur was also observed. No other organ injuries were observed. Her initial serum hemoglobin level, platelet count, and % prothrombin time were 10.8 g/dl, 21.4 × 10^4^/μl, and 72.7% before fluid resuscitation, respectively. She was transferred to our hospital for surgical management.

During the emergent transportation by an ambulance, her hemodynamic condition was stable with fluid resuscitation and blood transfusion. Another 30 ml/kg of lactated Ringer’s solution and 560 ml of packed red blood cells (four units) were given. On arrival at our institution about 4 h after the injury, her hemodynamic condition became unstable. Her BP was 90/58 mmHg, HR was 126 beats per minute, and RR was 19 breaths per minute. Her body temperature (BT) was 34.6 °C, and SatO_2_ was 100% under a 10-L oxygen mask. Her GCS was 15. She was pale, and her abdomen was remarkably distended. Because she was hemodynamically unstable, we added another 840 ml of packed red blood cells (six units). Despite aggressive hemodynamic resuscitation, her vital signs were still unstable. Abdominal compartment syndrome became apparent. At this time, her serum hemoglobin was 15.9 g/dl, and the platelet count was 1.7 × 10^4^/μl. The coagulation panel of the prothrombin time international normalized ratio was abnormal at 2.78, and her fibrinogen level had decreased to 34 mg/dl. Her acidosis progressed to a pH of 7.131, HCO_3_^-^ of 13.6 mmol/l, BE of −15.08 mmol/l, and lactate of 7.2 mmol/l. Moderate transaminitis with aspartate aminotransferase of 223 U/l and alanine aminotransferase of 163 U/l were also found. The total bilirubin level was 0.5 mg/dl, and the alkaline phosphatase level was 193 U/l.

**Fig. 2:**
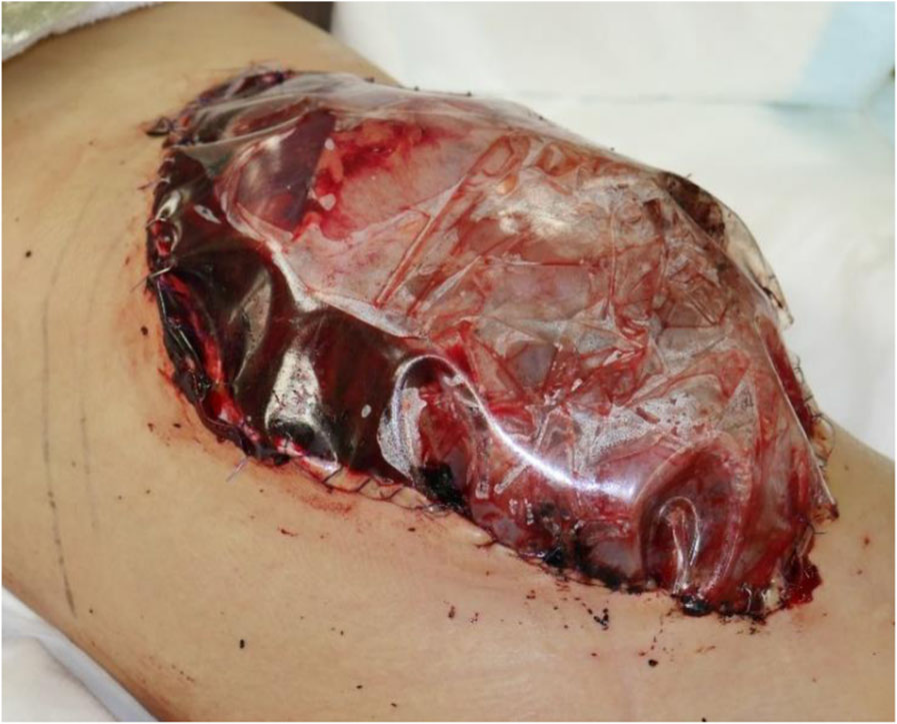
The patient’s abdomen after damage control surgery. Venous hemostasis was achieved by direct surgical gauze packing, and then the abdomen was closed temporarily using a plastic sheet to cover it

Because her condition had deteriorated and the lethal triad of low BT, coagulopathy, and acidosis was observed, a DC treatment strategy was selected. We planned to perform perihepatic gauze packing first and then immediately perform TAE with interventional radiology. After rapid intubation in the emergency room, she was transferred to the operating room. A 7-Fr intra-aortic balloon occlusion catheter was inserted via the left femoral artery before opening the abdomen to stabilize the general circulation. The liver was ruptured and a deep laceration was observed between the right anterior and the right lateral section of the liver after opening the abdomen. The posterior branch of the portal vein and the right hepatic artery was injured. The right hepatic vein was also injured and bleeding severely. We performed perihepatic gauze packing using Pringle’s maneuver with intermittent inflation of the intra-aortic balloon to maintain adequate blood pressure. The venous bleeding was controlled by direct surgical gauze packing, but the arterial bleeding continued. We placed drainage tubes into the abdominal cavity. Since the intestine was edematous and distended, primary closure of the abdominal wall was impossible. We therefore covered the abdomen using a plastic sheet (Fig. 2). The operative time was 91 min, total blood loss was 1500 ml, and the total blood transfused during the operation was 560 ml of red blood cells (four units), 960 ml of freshly frozen plasma (eight units), and 400 ml of platelets (ten units × 2).

**Fig. 3:**
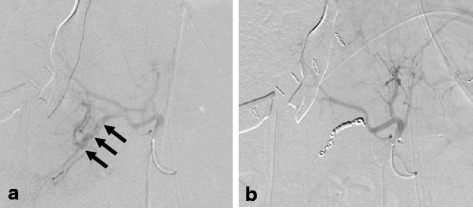
**a** Hepatic angiography immediately after perihepatic packing shows extravasation arising from a posterior branch (*arrows*) of the right hepatic artery. **b** Hepatic angiography after coil embolization shows the complete cessation of extravasation and a preserved patency of the anterior branch of the right hepatic artery

Immediately after perihepatic packing, she was transferred to the interventional radiology suite for diagnosis and treatment (about 8 h after injury). Acidosis (pH 7.238), coagulopathy (% prothrombin time 18%), and low BT (34.0 °C) were still observed. The radiologists were ready to perform TAE. A 5-Fr vascular catheter was inserted via the right femoral artery. Superior mesenteric arteriography revealed poor right portal venous flow. Celiac arteriography revealed extravasation from a posterior branch of the right hepatic artery (Fig. 3a). The posterior segmental branch was successfully embolized with gelatin sponge particles and nine hypersoft detachable helical coils (Fig. 3b). Complete arterial hemostasis was achieved and confirmed by hepatic arteriography. The TAE procedure time was 125 min. A total of 720 ml of freshly frozen plasma (six units) and 200 ml of platelets (ten units) was given during TAE.

**Fig. 4:**
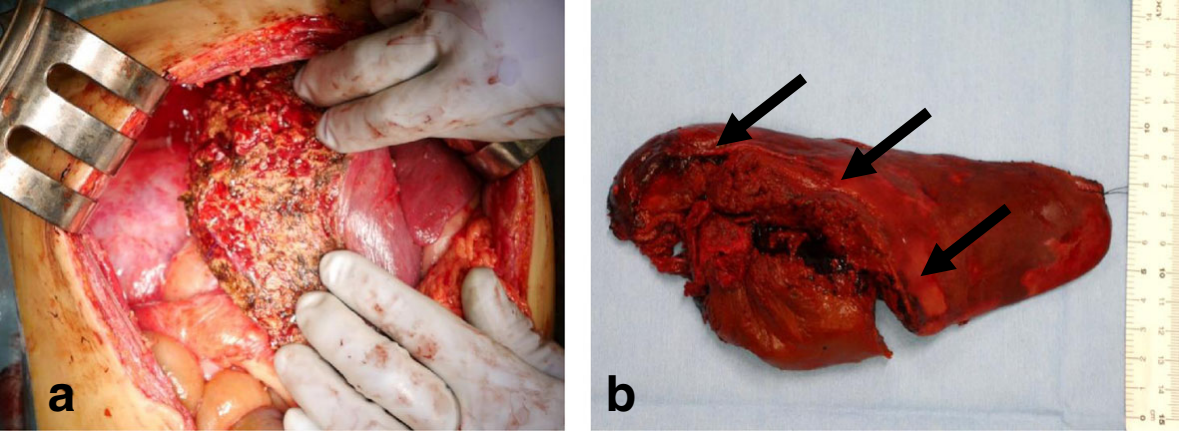
**a** Operative view immediately after delayed hepatectomy. Extended right lateral sectionectomy was performed after 48 hours of perihepatic packing. **b** A surgical specimen of the delayed hepatectomy. The excised liver was completely necrotic macroscopically and deep liver laceration was observed (*arrows*)

The patient was transferred to the intensive care unit (ICU). In order to stabilize her hemodynamic condition, she was treated with re-warming, intravenous fluids, and bed rest. On admission to ICU, her BP was 120/78 mmHg, HR was 132 bpm. Acidosis (pH 7.257), coagulopathy (% prothrombin time 34%), and low BT (35.9 °C) were still observed. Serial hemoglobin measurements revealed no reduction in the hemoglobin level. Her hemodynamic state was stabilized gradually. Her pH was 7.418, % prothrombin time was 51%, and BT was 38.5 °C at 48 h after medical treatment in ICU. Acidosis, coagulopathy, and low BT were all improved. At 48 h after perihepatic packing and TAE, we decided to perform re-laparotomy and delayed hepatic resection. There was two reasons to perform second operation. The first reason was the necessity of removal of the packed gauze and the closure of the abdomen to avoid infectious complications. The second reason was hepatic necrosis after TAE. Hepatic necrosis is high risk of hepatic abscesses. In our institute, hepatic necrosis is the indication of hepatectomy for successfully bleeding arrested cases after damage control surgery. We confirmed no portal flow of the posterior branch by the doppler ultrasonography before second operation. We gently removed the gauze for perihepatic packing and irrigated the abdominal cavity. We carefully confirmed hemostasis of the liver and the presence of necrotic lesions in the segments VI and VII. We performed extended right lateral sectionectomy including the necrotic area and laceration of the liver using Pringle’s maneuver (Fig. 4a, b). The histopathological examination of the liver specimen showed the massive necrosis of the liver. The intestinal edema and distention were improved. Those findings allowed us to perform direct closure of the abdominal wall. The total ischemic time of the whole liver was 30 min, the operative time was 251 min, the total blood loss was 240 ml, and the intraoperative blood transfusion was 280 ml of red blood cells (two units) and 240 ml of fresh frozen plasma (two units).

**Fig. 5:**
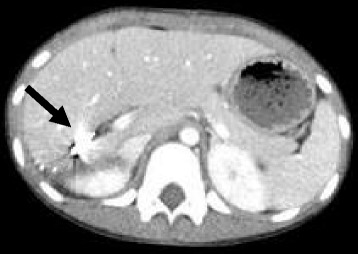
An axial view of an enhanced abdominal computed tomography (CT) scan on the 14th day after extended right lateral sectionectomy shows no evidence of an intra-abdominal abscess and biloma. Embolized coils (*arrow*) are also observed

The patient was returned to the ICU and received intensive care. Her postoperative course was uneventful. Her left trochanteric fracture was surgically treated with open reduction and internal fixation on day 5 after admission. Her total ICU stay was 10 days. A follow-up abdominal CT scan with contrast showed sufficient portal and hepatic arterial flow without extravasation, abscess formation, or biloma on day 14 (Fig. 5) and day 28 after the delayed hepatic resection. She was transferred to the local hospital to continue physical rehabilitation on day 42 after admission. She was discharged from the local hospital and went home on day 69 after the onset of her blunt liver injury. She had returned to her normal life by 3 months after the injury.

## Discussion

Abdominal trauma is a frequent cause of morbidity and mortality in children. Liver injury occurs in 15–20% of all pediatric patients with abdominal trauma [6]. The management of pediatric liver injuries has evolved over the last three decades [7], with decreasing rates of operative intervention. Currently, nonoperative management is considered to be the standard care in children suffering from blunt solid organ injury who are in a hemodynamically stable condition, with success rates exceeding 90% [8]. However, pediatric patients in hemodynamically unstable conditions due to blunt solid organ injury are considered candidates for operative intervention. In such critical situations, the DC strategy has proven useful for life-saving maneuvers.

The principles of DC in children are the same as in adults. *Damage control* was reported by the group at the University of Pennsylvania in 1993 [9]. They described the initial control of hemorrhaging by gauze packing and decontamination by saline irrigation of the abdominal cavity. After temporal closure of the abdominal wall with a plastic sheet, further intensive care should be done in the ICU, and delayed definitive repair of intra-abdominal injuries should be performed. A rapid transfusion of a large volume of resuscitation fluid to maintain stable vital signs with massive hemorrhaging results in coagulopathy, known as *the bloody vicious cycle*. This is statistically correlated with hypothermia and acidosis, and the three signs of coagulopathy, hypothermia, and acidosis constitute *the deadly triad* [10]. In the present case, the patient showed hemorrhagic shock with hypothermia, acidosis, and coagulopathy. She was also diagnosed with abdominal compartment syndrome due to massive intra-abdominal fluid collection. For these reasons, we adopted a DC strategy.

Temporary gauze packing in DC surgery has been reported to be useful in pediatric patients [11]. Stylianos reported 22 children 6 days to 20 years of age in whom intractable bleeding from the liver was controlled by gauze packing, and 90% of these cases were associated with coagulopathy, hypothermia, and acidosis. Packing was reported to be useful for controlling hemorrhaging in 95% of the patients. Temporal abdominal closure was required in 45% of the patients, and 82% of the patients survived [11]. We also selected temporal abdominal wall closure with a plastic sheet in the present case, because the edematous intestine was markedly dilated.

TAE of hepatic arterial injuries has been used for hemodynamically stable adult patients as an adjunct procedure of nonoperative management. TAE has also been recommended as a treatment for bleeding after DC surgery [12]. This procedure is currently widely accepted, and the success rate has been reported to be 83–100% [13–15]. In children, only a few cases of TAE for blunt hepatic injury have been reported, all of whom are still alive at the time of filing [6, 16–22]. In a review of TAE used in hemodynamically stable trauma pediatric cases, the complication rates ranged from 0 to 4% and were mostly minor troubles such as puncture site hematoma [16]. In the present case, the application of TAE after gauze packing hemostasis of venous bleeding appeared to be effective for controlling arterial bleeding from the injured liver. Although the patient developed hepatic necrosis after TAE, this complication was expected and is a common complication after TAE in adults, especially with high-grade liver injury. Dabbs et al. reported that 42.2% of patients went on to develop a major hepatic necrosis after TAE, and 96.7% of patients with a major hepatic necrosis needed operative intervention [23]. In our institute, hepatic necrosis is the indication of hepatectomy for successfully bleeding arrested cases after DC surgery. Delayed hepatic resection for the necrotic injured liver after DC surgery and TAE is well established in adults [24]. However, there have been no reports of pediatric cases. Li Petri et al. reported their excellent results suggesting that delayed hepatic resection may be an option in select cases at institutions with the appropriate expertise [24]. In the present case, TAE of the selected artery proved useful for reducing the area of liver necrosis and minimizing the operative insult of liver resection. Delayed hepatic resection was accomplished safely without any complications.

## Conclusions

The DC strategy was found to be effective even in a pediatric patient with hemodynamically unstable severe blunt hepatic injury. The presence of the deadly triad (hypothermia, coagulopathy, and acidosis) and abdominal compartment syndrome was an indication for this patient to undergo DC surgery. A correct and early decision regarding surgery is necessary to achieve a good outcome. Collaborative teamwork with emergency physicians, anesthesiologists, interventional radiologists, and liver surgeons will further improve the usefulness of DC surgery.

## Abbreviations

BP: Blood pressure
BT: Body temperature
CT: Computed tomography
DC: Damage control
GCS: Glasgow coma scale
HR: Heart rate
ICU: Intensive care unit
RR: Respiratory rate
SatO_2_: Percutaneous oxygen saturation on the index finger
TAE: Transarterial embolization
